# Attraction and achievement as 2 attributes of gamification in healthcare: an evolutionary concept analysis

**DOI:** 10.3352/jeehp.2024.21.10

**Published:** 2024-04-11

**Authors:** Hyun Kyoung Kim

**Affiliations:** /Department of Nursing, Kongju National University, Gongju, Korea; Hallym University, Korea

**Keywords:** Delivery of health care, Gamification, Health care outcome assessment, Health personnel, Information services

## Abstract

This study conducted a conceptual analysis of gamification in healthcare utilizing Rogers’ evolutionary concept analysis methodology to identify its attributes and provide a method for its applications in the healthcare field. Gamification has recently been used as a health intervention and education method, but the concept is used inconsistently and confusingly. A literature review was conducted to derive definitions, surrogate terms, antecedents, influencing factors, attributes (characteristics with dimensions and features), related concepts, consequences, implications, and hypotheses from various academic fields. A total of 56 journal articles in English and Korean, published between August 2 and August 7, 2023, were extracted from databases such as PubMed Central, the Institute of Electrical and Electronics Engineers, the Association for Computing Machinery Digital Library, the Research Information Sharing Service, and the Korean Studies Information Service System, using the keywords “gamification” and “healthcare.” These articles were then analyzed. Gamification in healthcare is defined as the application of game elements in health-related contexts to improve health outcomes. The attributes of this concept were categorized into 2 main areas: attraction and achievement. These categories encompass various strategies for synchronization, enjoyable engagement, visual rewards, and goal-reinforcing frames. Through a multidisciplinary analysis of the concept’s attributes and influencing factors, this paper provides practical strategies for implementing gamification in health interventions. When developing a gamification strategy, healthcare providers can reference this analysis to ensure the game elements are used both appropriately and effectively.

## Graphical abstract

[Fig f3-jeehp-21-10]

## Introduction

### Background/rationale

Digital innovations have technologically revolutionized health interventions and health education, driving a trend during the coronavirus disease 2019 pandemic toward the use of game elements to engage audiences both virtually and in person, reinforcing knowledge, behaviors, and attitude changes [1]. Gaming elements have facilitated internet-based interventions to connect with audiences in diverse ways, employing virtual reality, augmented reality, chatbots, and artificial intelligence [2]. Even post-pandemic, the appeal of video training and internet interventions has not waned, and these modalities have remained attractive for both audiences and providers [3]. This application of game elements in education, known as gamification, has seen extensive use in the healthcare field [4]. However, due to a lack of understanding among health professionals, gamification strategies are underutilized in the development of mobile applications, virtual reality interventions, and web-based interventions [5]. Furthermore, confusion between the concepts of gamification and serious games has impeded the implementation of true gamification elements, obstructed professional collaboration, and slowed knowledge development [6].

Since the application of gamification in healthcare is a relatively recent development, The evolutionary method by Rogers and Knafl [7] is well-suited to capture the dynamic nature of its meaning. This method differs from other types of conceptual analyses in that it emphasizes the evolution of the concept, characterized by a comprehensive and systematic review of the literature. The analytical process is philosophically driven, employing a deductive method of inquiry to delve into the essence of the concept. One of the strengths of this type of conceptual analysis is its ability to incorporate the context of the concept over time, thereby aiding further theoretical development. A unique feature of this conceptual analysis is its discouragement of case creation, as the application of concepts can vary across time and context, rendering cases unsuitable for exploring the concept’s nature [7]. However, this conceptual analysis method does have its drawbacks. It does not examine a large body of literature to evaluate its quality, nor does it provide guidance on how to integrate qualitative and quantitative data [8].

The steps involved in evolutionary conceptual analysis are as follows: (1) identifying the concept of interest along with surrogate terms; (2) conducting a literature search across various disciplines to analyze the concept’s context; (3) identifying the attributes (characteristics with dimensions and features), antecedents, influencing factors, surrogate terms, related concepts, and consequences, which collectively define the concept; (4) systematically analyzing the literature until content saturation is achieved; (5) identifying qualitative literature to provide exemplars that clearly illustrate the attributes, antecedents, and consequences of the concept (this step is optional); and (6) interpreting the results to identify implications and hypotheses. These 6 steps are not sequential, but rather simultaneous and cyclical [7]. The conceptual analysis of the hybrid model [8], as well as Walker and Avant’s work [9,10], was primarily conducted in the context of the nursing metaparadigm: human, environment, health, and nursing.

This study utilized the evolutionary method, which has been recently adopted and described in health interventions and integrates various disciplines to analyze innovative concepts [7].

### Objectives

The aim of this study was to analyze the concept of gamification in healthcare using the evolutionary method. The research question was: “What are the attributes, antecedents, influencing factors, surrogate terms, related concepts, consequences, implications, and hypotheses of gamification in healthcare?” This study aims to guide health professionals in applying gamification to their audiences and evaluating their responses.

## Methods

### Ethics statement

This study analyzed existing literature and did not include any human participants. Therefore, neither the institutional review board’s approval nor obtaining informed consent was required.

### Study design

This review study was conducted using the evolutionary concept analysis method by Rogers and Knafl [7].

### Identifying literatures

A literature review was conducted from August 2 to August 7, 2023, utilizing electronic databases such as PubMed Central, the Institute of Electrical and Electronics Engineers (IEEE), the Association for Computing Machinery (ACM) Digital Library, the Research Information Sharing Service (RISS), and the Korean Studies Information Service System (KISS). The search terms were derived from MeSH (Medical Subject Headings) terms and natural language, using the keywords “gamification and health*” or “healthcare.” The advanced search was specifically tailored to retrieve peer-reviewed articles and journal publications in either English or Korean, with full-text availability.

### Inclusion and exclusion criteria

The inclusion criteria were as follows: (1) articles related to gamification and healthcare for clients; (2) quantitative, qualitative, or review studies; and (3) articles written in either English or Korean. The exclusion criteria comprised: (1) articles unrelated to serious games as defined by the use of games in their entirety [6]; (2) articles unrelated to entertainment games for entertainment or fun [6]; (3) articles concerning education for students or healthcare providers; and (4) works in progress, conference presentations, dissertations for degrees, and theses.

### Literature selection

**Fig. 1.**
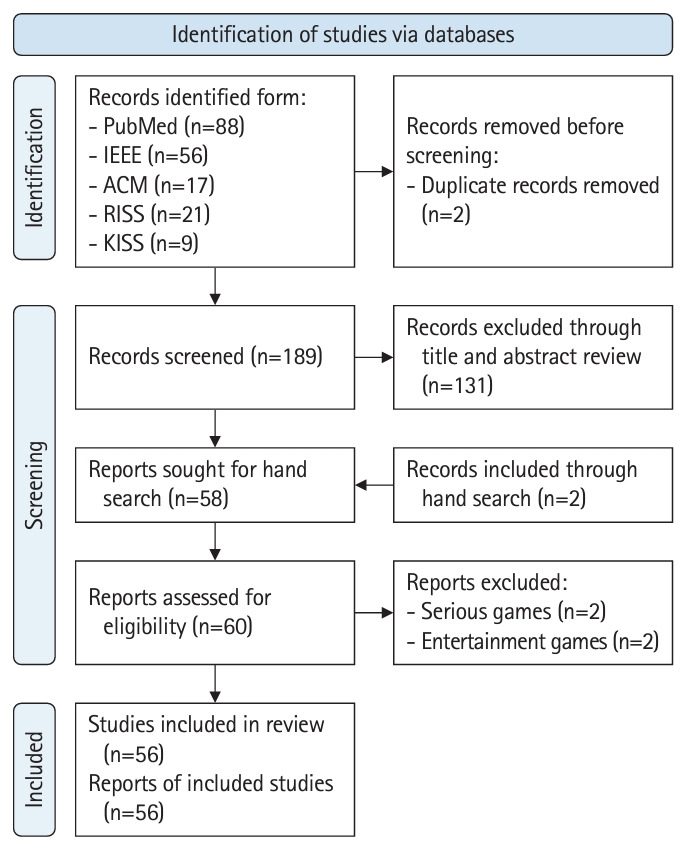
Literature search flow. IEEE, Institute of Electrical and Electronics Engineers; ACM, Association for Computing Machinery; RISS, Research Information Sharing Service; KISS, Korean Studies Information Service System.

In total, 68 articles were scrutinized from various databases: 43 out of 88 articles identified from PubMed Central, 5 out of 56 from IEEE, 4 out of 17 from the ACM Digital Library, 1 out of 21 from RISS, and 1 out of 9 from KISS. These were selected by the author, who screened titles and abstracts. The publication dates of these articles ranged from 2014 to 2023. Two additional studies were added from the reference lists through a manual search. Two articles were removed due to duplication, and 4 articles—2 related to serious games and 2 concerning education for nursing students and dentists—were extracted from the lists after a full-text reading. Ultimately, 56 articles were included in the concept analysis (Fig. 1, Supplement 1).

### Data extraction

**Fig. 2.**
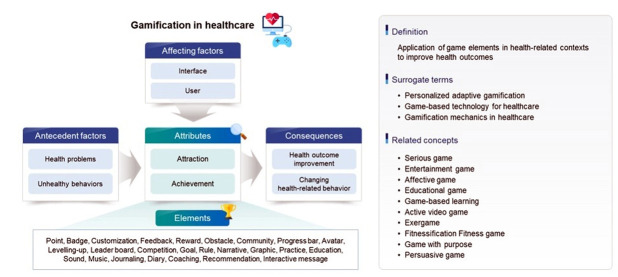
Diagram of results of the concept analysis for gamification in healthcare according to Rogers’ evolutionary analysis.

Data extraction was conducted in accordance with the protocol of the evolutionary method to ensure consistency and reliability. The items for extraction were predetermined, gathered, and documented in the case report note. These items included the first author, publication year, country, discipline, study design, healthcare domain, game type, health outcomes, target concepts, attributes, antecedents, influencing factors, surrogate terms, related concepts, consequences, implications, and hypotheses. A professional in healthcare informatics reviewed the extracted notes for an external audit and credibility check. The expert’s feedback was incorporated, revisions were made, and the changes were validated and confirmed through email and meetings. Finally, the collected data were summarized and reported, and the categorized items were visually represented in graphics using themes (Fig. 2).

### Statistical methods

Descriptive statistics were used to present the results.

## Results

### General characteristics of the literature

Publications from 2014 to 2023 showed that the United States had the highest number of first authors (n=17), followed by Australia (n=5), Spain (n=5), Germany (n=4), and other countries. The academic disciplines represented were primarily medicine (n=13), health science (n=11), engineering (n=10), and informatics (n=3), among others. The study designs included reviews (n=31), randomized controlled trials (RCTs) (n=11), RCT protocols (n=4), and other designs. The healthcare domains covered were mental care (n=8), obesity (n=7), perception disability (n=4), and others. The types of games used were predominantly mobile applications (n=21), whereas fewer studies used social media (n=2), virtual reality (n=2), and other platforms. The health outcomes studied included activity (n=6), diet (n=3), depression (n=3), memory (n=3), and others.

### Definitions of gamification in healthcare

Gamification is defined as a comprehensive, non-entertainment platform that, while not a game in its own right, employs elements of a game and utilizes game design and game elements in non-game contexts [11]. It is a strategy aimed at influencing user behavior and motivation through game-like experiences [12]. This approach seeks to increase participant usage by applying game-like mechanisms to real-world interactions [13]. Essentially, it involves the use of game-like elements in non-game contexts to boost user engagement and motivation [14]. In summary, when applied to healthcare, gamification refers to the use of game elements in health-related contexts to improve health outcomes (Fig. 2).

### Attributes of gamification in healthcare

The attributes of gamification in healthcare encompass a variety of elements, including an attraction to health behaviors and the achievement of health-related goals. These elements include dynamics, mechanics, specifics, and aesthetics [11]. They also involve engagement, motivation, personalization, and entertainment [15]. User experience, immersion, dynamic capture affection, performance, and accuracy are also key attributes [16]. The list continues with challenge, fantasy, and curiosity [17], as well as professionalism, relationship, autonomy, bonding, trigger, action, and investment [18]. Metaphors, social modeling, and framing are also included [19]. Finally, fun, esteem, growth, sustainability, self-representation, socializing, and self-managing are also integral components of gamification in healthcare [20] (Fig. 2).

### Antecedents of gamification in healthcare

The antecedent factors of gamification in healthcare include various health problems such as stroke [15], diabetes mellitus [21], cognitive disability [18], depression [22], cancer [23], obesity [24], attention deficit hyperactivity disorder [25], and fractures [26]. Additionally, unhealthy behaviors like smoking [27], lack of physical activity [28], and an unhealthy diet [24] also serve as antecedent factors (Fig. 2).

### Factors influencing gamification in healthcare

The factors influencing gamification in healthcare can be categorized into interface and user components. The interface components include purpose, content, audience, economics, application area, sociality, technology [29], guidelines, and narrative [30]. The user components encompass participation [30], user preference, intention [31], attention, knowledge, problem-solving competency, memory, and strategy [12] (Fig. 2).

### Surrogate terms for gamification in healthcare

Personalized adaptive gamification, or game-based technology for healthcare, shares similarities with broader concepts of gamification [16]. Gamification mechanics in healthcare can also be viewed from the perspective of pedagogy—that is, engagement with learning mechanisms in appealing, user-centric alternatives (Fig. 2).

### Concepts related to gamification in healthcare

A “serious game” is defined as a game that does not primarily aim for entertainment, enjoyment, or fun [32]. These games are designed for non-entertainment purposes across various sectors such as the economy, education, health, industry, military, and politics. The distinction between a serious game and gamification lies in the fact that gamification serves as an instrumental function of a real-world system, whereas a serious game is a concept that exists independently of the system [6]. Whereas serious games are purposes in themselves, gamification involves the use of game elements in non-game contexts for the purposes of learning, teaching, or solving problems [6]. There are numerous related concepts. For instance, an exergame is an intervention that leverages the aesthetics and fun of video games to make physical activity appealing, thereby encouraging users to continue playing [13]. An entertainment game, on the other hand, is a game that seeks to entertain for its own sake. An affective game is an intervention that acknowledges the user’s emotional state to modify the game configuration and improve the gaming experience [16]. Other concepts similar to gamification include educational games, game-based learning, active video games, fitnessification, fitness games, games with purpose, persuasive games, and persuasive information systems in healthcare [6] (Fig. 2).

### Outcomes of gamification in healthcare

The primary outcomes of gamification in healthcare include improvements in health outcomes [33] and modifications in health-related behavior [21]. Additional outcomes encompass recovery, rehabilitation, implementation of practice, mobility, reduction in hospitalization, and self-management [15]. Gamification also enhances users’ competencies in problem-solving, understanding, memory, and concentration [12,31], as well as strategic thinking and goal orientation [12]. It also aids in maintaining long-term adherence, attention [24], and provides entertainment and social communication [33]. Furthermore, it facilitates the completion of tasks and performance [16] (Fig. 2).

## Discussion

### Key results

This concept analysis study was based on the author’s hypothesis that users could achieve their desired health outcomes more effectively through the use of gamification mechanics. This study has implications for healthcare providers who deliver services via mobile applications, web-based healthcare, artificial intelligence chatbot interventions, virtual reality, augmented reality, or social network services. The first core attribute of gamification in healthcare is the ability to attract and motivate users, encouraging them to participate, engage, immerse themselves, adhere to, and maintain health behaviors. Aesthetic elements, including visual frames, game visual design, avatars, music, sound, and metaphors, play a role in this process. The second attribute of gamification in healthcare is achievement. Unlike other strategies, gamification employs unique methods such as challenges, badges, leaderboards, rewards, and entertainment.

### Interpretation

Healthcare providers seek efficient interventions that improve health outcomes for their patients, but users may disengage from these interventions, perceiving them as tedious obligations. Gamification has unveiled a new horizon that meets the needs of both health professionals and users by leveraging entertainment as a primary motivator within the health context. Traditional learning theories often bore users, leading to mechanization and the fragmentation of the user experience. In contrast, games provide entertainment, enriching users emotionally and aesthetically.

Customized health intervention games provide individualized care solutions. In the realm of virtual reality, for instance, individuals undergoing stroke rehabilitation have the flexibility to choose and advance in exercises aimed at increasing their limb mobility, with the additional incentive of earning badges and engaging in competitive games to receive trophies for outstanding achievements, thereby markedly improving their physical health outcomes. Similarly, younger individuals have the opportunity to strengthen their emotional well-being by designing personal avatars and partaking in immediate interactions with counterparts to address issues of depression and social isolation in an aesthetically pleasing setting. Elderly individuals experiencing mild dementia can participate in mini-games designed with straightforward cognitive and visual puzzles, with a progress bar to monitor their advancements. In the sphere of educational settings, augmented reality simulations pertaining to patient safety management enable medical students to significantly improve their retention of learning, knowledge acquisition, skill development, and professional demeanor by navigating through incidents such as medication discrepancies, transfusion mishaps, and slip-and-fall accidents.

Nonetheless, certain drawbacks require attention. The promotion of gamification applications may lead to a decline in face-to-face interactions among youth, reducing complex human cognitive processes to basic elements of gaming. This approach might incite undue competitive spirit for achievements, further intensifying the competitive ethos prevalent in societies such as that of Korea. Challenges associated with personal data security, the financial burdens of game creation, and the prerequisites of internet connectivity and digital proficiency might exacerbate existing health inequities.

To counteract these concerns, the incorporation of social networking, peer encouragement, and mentorship programs is essential to re-establish the social engagements often missing in gaming experiences. In gamification efforts, it is imperative to eschew methods that provoke negative emotions, boredom through repetitive tasks, undue commendation, poorly structured interventions, or unexpected assignments. Employing “nudges” as a gamification tactic can capitalize on the natural human inclination to avoid losses, motivating individuals to prioritize long-term health advantages over short-term indulgences. Therefore, rather than limiting gamification to mere leisure and accomplishment, it is more beneficial to adopt a comprehensive and dynamic approach to digital platform integration. Ideally, gamification initiatives should aim to be therapeutic, encourage personal development, and nurture positive encounters without impeding the autonomy of choice.

### Comparison with previous studies

The 10 elements of gamification include points, badges, customization, feedback, rewards, obstacles, community, progress bars, avatars, and leveling-up [17]. These elements can be implemented using various strategies such as leaderboards, competition, achievements, goals, rules, narratives, and graphics [21]. Additional strategies include tutorials, voting, mentoring [18], practice, education, sound, music, journaling, diaries, daily planning, coaching, recommendations, and interactive messaging [34]. Other strategies encompass prompts, push alarms, feedback [35], characters, plots, control sticks, graphics [30], steps, scores, stickers, awards, stars, and chat features [34]. Monitoring, congratulatory emails [36], nudges, reminders, and social modeling [19] are also effective strategies.

Strategies to enhance gamification elements involved using association, reward-threat dynamics, and goal-planning, as well as changing taxonomy [36]. It also included presenting rules and fostering interaction [33]. Other elements were characterizing visual appeal, ensuring ease of use, promoting learning through action, and maintaining interest [37]. Additionally, strategies aimed at increasing socialization, recognition, reciprocal benefits, and network exposure [38]. Enhancing simplicity, readability, and hierarchy was also key [18], as well as providing guidelines, narratives, interfaces, and opportunities for participation [30].

Personalization can be utilized in healthcare gamification through methods such as goal setting, user targeting, inter-human interaction, adaptation, content awareness, and self-learning [39]. Additionally, reminders, alerts, and financial incentives can be employed [40]. Personalization serves as a core intrinsic motivator, fostering self-esteem and personal growth [20]. It can be implemented in gamification through the use of avatars as self-representatives, autobiographical storylines, and emotional design [41]. Sophisticated gamification users are often more likely to adhere to healthcare programs. Socialization, facilitated through competition, collaboration, and social support [5], can be a key factor in achieving satisfactory user experiences. Social networking promotes participation and communication among users. Real-time feedback from healthcare professionals can prevent demotivation in monotonous situations and encourage long-term engagement [12].

Understanding gamification in healthcare helps healthcare professionals understand how to design interventions. A mobile application for pregnant women has been developed to improve environmental health behavior and prevent exposure to risk factors [42]. This application provides a clear goal, guidelines for use, and a reward system that includes a leaderboard to motivate competition. Users can easily view their achievement badges, and the application features an attractive, personalized avatar to foster a sense of ownership. The content is tailored to women of advanced maternal age, and feedback is provided by a midwife consultant. The ability to chat with other users further improves health behavior and health outcomes.

### Limitations/strength

This study had several limitations. The literature review was confined to English and Korean languages, and only articles from peer-reviewed journals were included in the data analysis. While multidisciplinary subjects were incorporated, the academic background of the researcher may have influenced the conceptual analysis. The strength of this study lies in its timely relevance, particularly in the context of the growing trend of online interventions aimed at changing health behaviors and improving health outcomes.

## Conclusion

The study meticulously explored gamification in healthcare, delineating its intrinsic attributes with a particular emphasis on attraction and achievement, and further enriching this analysis by introducing 2 additional dimensions: enhanced attraction and deepened achievement. Through the lens of Rogers’ evolutionary concept analysis, the research delineates the transformative potential of gamification to augment health interventions, underscoring the significance of embedding game elements not only to engage patients but also to fundamentally enhance their healthcare journey. Healthcare providers and professionals can utilize clear definitions, antecedents, attributes, and consequences to broaden primary care, treatment, and rehabilitation across various health domains, including physical, emotional, social, psychological, and spiritual. Gamification holds the potential to revolutionize healthcare by improving user engagement, facilitating more effective education, and promoting behavioral change.

## Figures and Tables

**Figure f3-jeehp-21-10:**
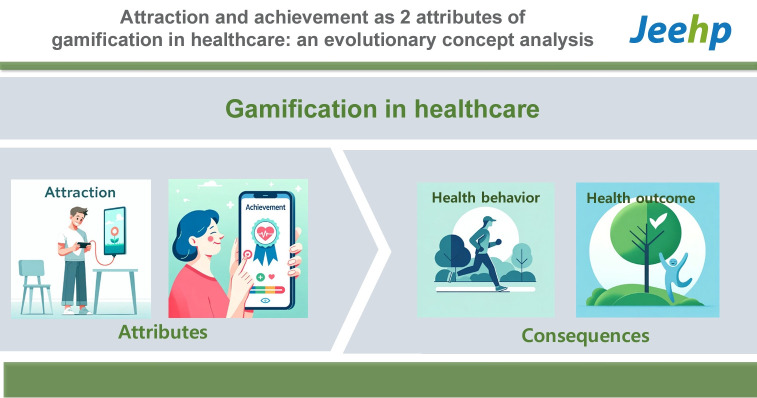


## Supplementary materials

Supplementary files are available from Harvard Dataverse: https://doi.org/10.7910/DVN/NBDNKH

Supplement 1. The 56 selected articles for the evolutionary concept analysis about gamification in healthcare.

Supplement 2. Audio recording of the abstract.
